# Associations of neck muscle strength and cervical spine mobility with future neck pain and disability: a prospective 16-year study

**DOI:** 10.1186/s12891-021-04807-3

**Published:** 2021-10-29

**Authors:** Juhani Multanen, Arja Häkkinen, Hannu Kautiainen, Jari Ylinen

**Affiliations:** 1 Faculty of Sport and Health Sciences, University of Jyväskylä, Jyväskylä, Finland; 2 Department of Physical Medicine and Rehabilitation, Central Finland Hospital, Jyväskylä, Finland; 3 Department of General Practice and Primary Health Care, University of Helsinki, Helsinki, Finland; 4 Unit of Primary Health Care, Kuopio University Hospital, Kuopio, Finland

**Keywords:** Neck pain, Neck disability, Association, Muscle strength, Range of motion

## Abstract

**Background:**

Neck pain has been associated with weaker neck muscle strength and decreased cervical spine range of motion. However, whether neck muscle strength or cervical spine mobility predict later neck disability has not been demonstrated. In this 16-year prospective study, we investigated whether neck muscle strength and cervical spine mobility are associated with future neck pain and related disability in women pain-free at baseline.

**Methods:**

Maximal isometric neck muscle strength and passive range of motion (PROM) of the cervical spine of 220 women (mean age 40, standard deviation (SD) 12 years) were measured at baseline between 2000 and 2002. We conducted a postal survey 16 years later to determine whether any subjects had experienced neck pain and related disability. Linear regression analysis adjusted for age and body mass index was used to determine to what extent baseline neck strength and PROM values were associated with future neck pain and related disability assessed using the Neck Disability Index (NDI).

**Results:**

The regression analysis Beta coefficient remained below 0.1 for all the neck strength and PROM values, indicating no association between neck pain and related disability. Of the 149 (68%) responders, mean NDI was lowest (3.3, SD 3.8) in participants who had experienced no neck pain (*n* = 50), second lowest (7.7, SD 7.1) in those who had experienced occasional neck pain (*n* = 94), and highest (19.6, SD 22.0) in those who had experienced chronic neck pain (*n* = 5).

**Conclusions:**

This 16-year prospective study found no evidence for an association between either neck muscle strength or mobility and the occurrence in later life of neck pain and disability. Therefore, screening healthy subjects for weaker neck muscle strength or poorer cervical spine mobility cannot be recommended for preventive purposes.

## Introduction

Neck pain is a highly prevalent condition that affects about two-thirds of the adult population at some time during the lifespan [1]. Women are affected more often than men, and it has been thought that one reason why neck pain is more common among women is that they have lower muscle strength than men [2]. Although maximal muscle strength is known to peak between ages 20 and 30 [3], evidence suggests that the prevalence of chronic neck pain peaks in middle-age and decreases thereafter [4, 5]. This is a puzzling finding that merits further investigation. Even though neck pain usually resolves within days or weeks, it has a high rate of transition to a chronic or persistent problem, as it becomes chronic in 5–7% of cases [6, 7]. The International Association for the Study of Pain (IASP) defines neck pain of more than 3 months’ duration as chronic [8]. Neck pain has substantial effects on quality of life and work ability, and thus imposes a significant personal and socioeconomic burden [9].

To prevent neck pain requires an understanding of the predisposing factors for its development. Although little is known about the etiology of neck pain and related disability, the literature suggests that the risk for developing neck pain may be affected by different physical, psychosocial, and individual-level factors [10]. In the case of physical risk factors, research has consistently shown an association between neck pain and decreased neck muscle strength [11–16]. Further, several randomized studies have reported a decrease in neck pain as a result of neck muscle-strengthening rehabilitation programs [17–20]. Similarly, patients with neck pain have shown a decreased cervical range of motion compared with persons without neck pain [11, 21–24]. Some randomized studies have also reported a decrease in neck pain and improved cervical range of motion as a result of neck muscle-strengthening rehabilitation programs [18, 25]. However, it remains unclear whether neck pain causes weakness in neck muscles or whether weak neck muscles generate neck pain. Similarly, the relationship between decreased spinal mobility and neck pain is equivocal.

It seems reasonable to assume that naturally good neck muscle strength and range of motion are likely to be protective factors against neck pain. Although numerous studies have investigated the association between neck pain and cervical muscle strength or range of motion, few prospective cohort studies have focused on the possible prognostic value of muscle strength and spine mobility for the later development of neck pain. The results of the few existing studies suggest that spine mobility has no predictive value for the later occurrence of neck pain in originally pain-free subjects, while the results for muscle strength have been conflicting [26, 27]. Thus, further evidence is needed to clarify whether neck muscle strength and cervical spine mobility could predict future neck pain. In addition, the previous studies have not taken chronic neck pain or related disability into consideration. The Neck Disability Index (NDI), which is the most commonly used self-report instrument for evaluating neck pain status, provides information not only about experiences of pain but also about a subject’s functional capacity [28]. Hence, in the present study we explored prospectively whether neck muscle strength and mobility of the cervical spine were associated with functional capacity in later life measured using neck pain and the NDI among subjects who were pain-free at the time of the baseline measurements.

## Methods

### Study design

This study was a 16-year prospective survey to assess the possible association of neck muscle strength and mobility with the development of neck pain and related disability. The Ethics Committee of the Central Finland Health Care District approved the study plan (protocol approval number 41/2000) which was designed in accordance with the guidelines of the Declaration of Helsinki. All participants gave their written informed consent prior to enrollment.

### Subjects

The original study group comprised female volunteers recruited through advertisements targeted to the personnel of the largest employers in the City of Jyväskylä, Finland. The study focused on females owing to the higher prevalence of neck pain in females [29]. The 241 subjects who indicated interest in the study were sent a screening questionnaire to assess their eligibility to participate. These individuals worked either for the municipality or at the local hospital or for various industrial facilities employing both blue- and white-collar workers, or they were students. The screening questionnaire included items on health status, occupation, and engagement in competitive sports. Inclusion criteria were being female, healthy, and aged between 20 and 59 years (this wide age range was purposely used to establish reference values for cervical spine mobility and muscle strength in working-age females). Exclusion criteria were: neck and shoulder pain experienced within the previous 6 months, previous or current injuries or other disorders of the neck or shoulder region, rheumatoid arthritis, fibromyalgia, severe depression or mental disorder, or active participation in competitive sport. We excluded 18 of the 241 volunteers owing to neck or shoulder symptoms and 3 owing to missing information, thus yielding a sample of 220 healthy females for the study.

The participants completed a questionnaire on their health status, occupation, level of physical workload, and time spent on leisure-time physical activity. The visual analog scale (VAS, 0–100 mm) was used to check that they had experienced no neck pain during the week preceding the baseline measurements.

### Baseline measurements

The baseline measurements, including body height and mass, were performed between November 2000 and October 2002 for all participants by the same physiotherapist. Maximal isometric strength of the flexor, extensor, and rotator muscles of the cervical spine was measured with a specially designed neck strength measurement system (NSMS; Kuntoväline Ltd., Helsinki, Finland). The wall-attached system has two adjustable, rigid plates to stabilize the subject’s trunk. The subject’s chest and waist were tightly fastened to these plates with wide straps at the level of the iliac crest and above the inferior angle of the scapula. The subject was seated with hips and knees at 90° of flexion. The head was held in an upright neutral position. During the testing of flexion force, the subject was seated directly facing the device with a bar equipped with a force cell in contact with her forehead. During the recording of extension force, the subject turned 180°, so that her back ended up facing the device and the force cell was in contact with her occiput. Muscle strength for cervical spine rotation was measured with an overhead module consisting of four pads that were attached to both sides of the subject’s head. The subject’s head was secured in a neutral position by tightening all four pads at the same time. In addition, the subject’s chin was supported with a bar to avoid head movements. The axis of rotation was adjusted by centering the overhead module parallel to a vertical line with external auditory canals. The force cell was attached to the axis of the overhead module.

Neck strength was measured first for rotation, followed by flexion and extension. Two warm-up trials were performed, followed by three maximum-effort trials in each direction. The highest result in each direction was used in the analyses. The results of the baseline measurements for the original 220 subjects have been presented in detail elsewhere [30]. These strength measurements have been found to have good intratester reliability with intraclass correlation coefficient (ICC) values ranging from 0.87 to 0.96, depending on the direction tested [30].

Passive range of motion (PROM) of the cervical spine using a cervical measurement system (CMS; Kuntoväline Ltd., Helsinki, Finland) was measured in all three planes of motion: lateral flexion (frontal plane), axial rotation (horizontal plane) to both the right and left sides, and flexion-extension (sagittal plane). The CMS includes two gravity goniometers, a compass goniometer, and two fluid levels attached to a plastic frame. Movement in the different planes is shown by the goniometers and compass in increments of 2°. The intra-rater reliability of this measure has been found to be good, with ICC values ranging from 0.79 to 0.92 [31]. In their original study, Salo and colleagues (2009) chose to assess age-related changes and establish reference values for passive range of motion of the cervical spine in healthy working-age women, as no prior studies had reported reference values for this parameter. The results of the baseline PROM measurements for the original study group of 220 subjects have been reported in detail elsewhere [31].

### Postal survey 16 years after baseline measurements

We mailed a questionnaire package to the participants 16 years after the baseline measurements. They were asked whether they had experienced no neck pain at all, or neck pain occasionally for short periods of time or continuously for at least 3 months (i.e., chronic pain) since the baseline measurements. Items on illnesses, accidents, and surgical procedures, and visits to health care professionals, treatments, and medication due to neck pain were also included. We also asked the subjects about their overall perceived health, cigarette smoking, body mass and height, time spent on leisure-time physical activity, occupation, and level of physical workload. Participants also completed the Finnish validated version of the Neck Disability Index (NDI) [32], originally reported by Vernon and Mior [33], which is a functional status questionnaire containing 10 items asking about pain, personal care, lifting, reading, headaches, concentration, work, driving, sleeping and recreation. Each item is scored on a 0 to 5 rating scale, yielding a possible total score of 50. We multiplied the participants’ score by two to obtain percentage scores, as instructed by Fairbank et al. [34]. A score of 0% indicates no activity limitations and a score of 100% indicates complete activity limitation [33]. The NDI has been found to have good reliability and validity in individuals with neck pain [28]. The minimally clinically important change reported by patients was 5–10 points on a scale of 0–50 (10–20%) [35].

### Statistical methods

We present descriptive characteristics using means and standard deviations (SD) or counts with percentages, and frequency distribution. The normality of variables was evaluated graphically and with the Shapiro-Wilk W test. The participants were divided into two groups according to the incidence of neck pain. We compared the groups using independent samples t-test and analysis of co-variance. For the primary outcomes between-group differences are given in mean with their 95% confidence intervals. We set the α level at ≤0.05 for all tests. We used multiple linear regression analyses to evaluate the association between the NDI and baseline neck strength and PROM values using adjusted (age and body mass index) standardized regression coefficients (Beta). The Beta value is a measure of how strongly each variable influences the criterion (dependent) variable. Beta is measured in units of standard deviation. Cohen’s standard for Beta values above 0.10, 0.30 and 0.50 represent small, moderate and large relationships, respectively [36]. We analyzed the data using the STATA 14.1 statistical software package (StataCorp, College Station, TX).

## Results

Of the 220 subjects originally recruited for the study, 149 (68%) returned the 16-year follow-up questionnaire. Participants’ characteristics and neck muscle strength and cervical spine PROM values are shown in Table 1. Of these 149 subjects, 50 (34%) reported that they had not experienced neck pain at all (hereafter No pain group), 94 (63%) reported occasional neck pain (Occasional group), and 5 (3%) continuous neck pain for at least 3 months (Chronic group) during the past 16 years. The three groups were similar in all participant characteristics (*p* = 0.21 to 0.87).

**Table 1:** Current clinical characteristics, baseline neck muscle strength and cervical spine range of motion of the females (*N* = 149), who returned the questionnaire at the 16-year follow-up

Variable	Value
Age, years, mean (SD)	57.3 (11.4)
Height, cm, mean (SD)	165 (6)
Body mass, kg, mean (SD)	69.7 (12.0)
Body mass index, kg/m^2^, mean (SD)	25.4 (4.0)
Smoker, n (%)	15 (10)
Retired, n (%)	57 (38)
Leisure time physical activity, h/week, mean (SD)	6.1 (4.3)
Strength	
Extension, N, mean (SD)	192 (30)
Flexion, N, mean (SD)	74 (19)
Rotation, Nm, mean (SD)	7.9 (2.1)
Range of motion	
Sagittal, °, mean (SD)	166 (18)
Horizontal, °, mean (SD)	191 (19)
Frontal, °, mean (SD)	89 (15)

Over the previous 16 years, six subjects from the Occasional group reported neck injuries: two collisions and one a whip-lash injury after a car wreck, two workplace head injuries, and one a wakeboard fall. Clavicle fractures were reported by two subjects in the Chronic group, one of whom reported sustained fracture-related shoulder and neck pain. A total of 32 medical visits, 27 from the Occasional group and five from the Chronic group, were made due to neck pain, of which nine were further investigated with different diagnostic imaging modalities. One subject in the No pain group reported surgery to remove lymph nodes from the neck area as part of treatment for salivary gland cancer, and one subject from the Occasional group reported a thyroidectomy.

Twenty-six subjects in the Occasional group and five subjects in the Chronic group reported receiving treatments for neck pain over a 16-year period. The most commonly-used treatment modalities were massage (26 subjects), exercise therapy (12 subjects), thermo- and cryotherapy (six subjects), acupuncture (five subjects), and cervical spine mobilization (four subjects). Twenty-three subjects in the Occasional group and two subjects in the Chronic group reported taking painkillers for neck pain during the last 12 months. They had taken painkillers for an average of 19 (SD 41, range 2 to 200) days within the past year.

**Fig. 1:**
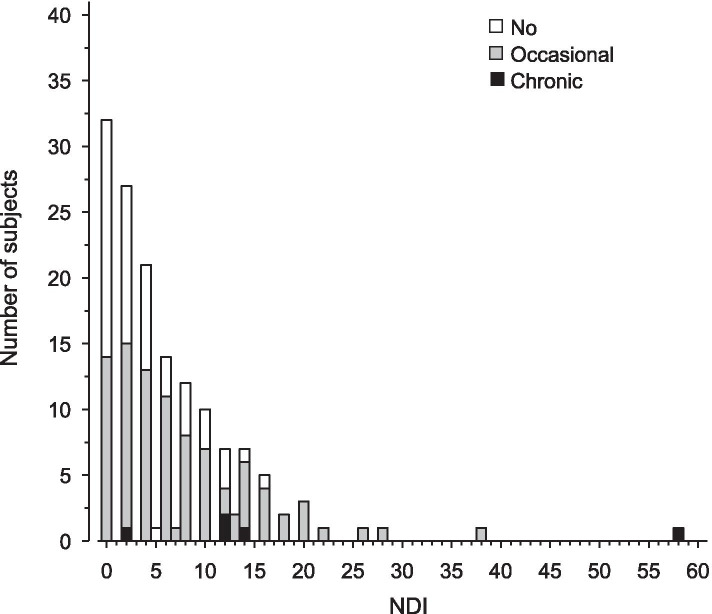
Distribution of neck disability indices in females who reported no neck pain, occasional neck pain or chronic neck pain during the 16-year follow-up

Mean NDI was lowest (3.3, SD 3.8, range 0 to 16) in the No pain group, second lowest (7.7, SD 7.1, range 0 to 38) in the Occasional group and highest (19.6, SD 22.0, range 2 to 58) in the Chronic group. The Chronic group had a significantly higher NDI than the No pain group (mean difference 16.3, 95% CI, 8.4 to 24.2) or Occasional group (11.9, 95% CI, 4.2 to 19.7). The NDI was also significantly higher in the Occasional group than in No pain group (4.3, 95% CI, 1.4 to 7.3). The distribution of NDI indices for the No pain, Occasional and Chronic subjects is shown in Fig. 1.

Since the Chronic group, which contained only 5 subjects, was too small to be included in any statistical test, the Chronic group data were pooled with the Occasional group data. Before pooling, it was ensured that the Chronic group did not differ from the Occasional group in any of the clinical characteristics (*p* = 0.20 to 0.83) or muscle strength (*p* = 0.22 to 0.99) or PROM values (*p* = 0.27 to 0.54). The pooled group is referred to hereafter as the Pain group. The No pain (*N* = 50) and Pain (*N* = 99) groups differed in age (mean 61.0, SD 10.9 years in the No pain and 55.5, SD 11.3 years in the Pain group, mean difference 5.5 years, 95% CI, 1.7 to 9.3). The Pain group had a significantly higher NDI than the No Pain group (mean 8.3, SD 8.7 in the Pain group and 3.3, SD 3.8 in the No Pain group, mean difference 4.9, 95% CI, 2.4 to 7.5).

**Fig. 2:**
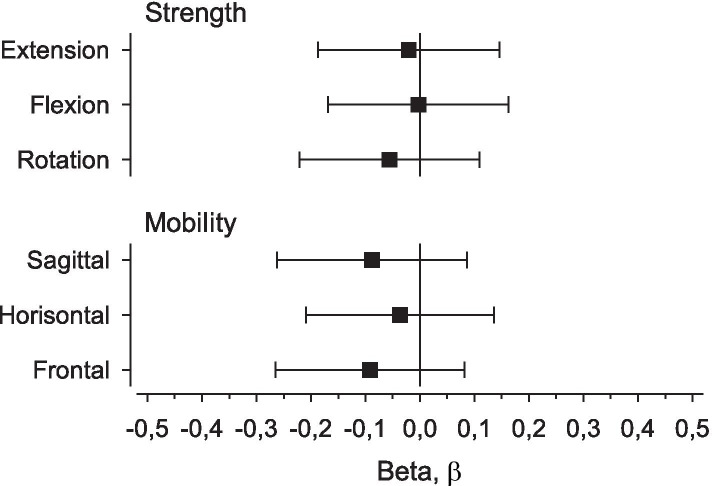
Association between Neck Disability Index (NDI) and neck muscle strength and cervical spine mobility using adjusted (age and body mass index) standardized regression coefficients (β). The NDI is plotted on the x-axis as β values with 95% confidence intervals

The analyses for the whole group (*n* = 149) revealed that none of the isometric neck strength measures in extension, flexion, or rotation were associated with neck disability. The β value for each of the independent predictors remained below − 0.1 (small) (Fig. 2). Similarly, none of the measures of passive mobility of the cervical spine in the sagittal, horizontal, or frontal planes were associated with neck disability, as all three β values were below − 0.1 (Fig. 2).

**Fig. 3:**
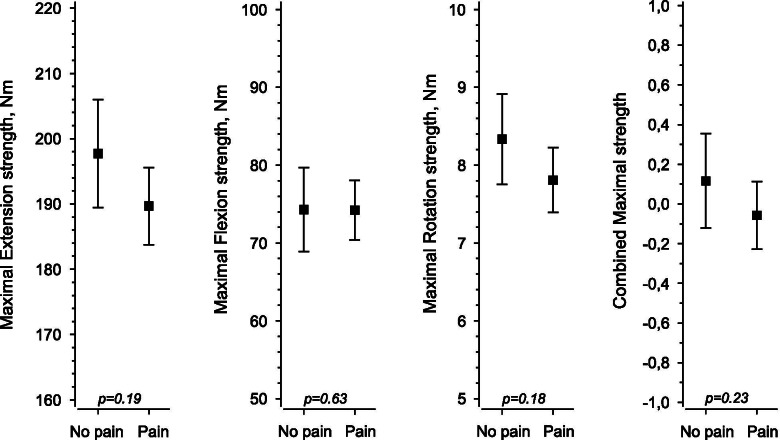
Neck muscle strength in women with no neck pain (No pain) and those with occasional or chronic neck pain (Pain) during the 16-year follow-up. The combined maximal strength values have been calculated from the average of the standardized values of maximal extension, flexion and rotation strength. Boxes show means and whiskers indicate 95% confidence intervals

The mean (95% CI) maximal extension strength, flexion strength, and rotation strength values were 198 (190 to 205) N, 74 (69 to 79) N, and 8.3 (7.7 to 8.9) Nm, respectively, in the No pain group and 190 (183 to 196) N, 74 (70 to 78) N, and 7.8 (7.4 to 8.2) Nm, respectively, in the Pain group (Fig. 3). No statistically significant differences were observed between the two groups in maximal extension strength (*p* = 0.19), flexion strength (*p* = 0.63), rotation strength (*p* = 0.18) or combined maximal strength (*p* = 0.23) (age-adjusted *p* values, Fig. 3). The strength values of the Chronic group did not differ from those of the No pain (*p* = 0.40 to 0.59) or Occasional groups (*p* = 0.61 to 0.99).

**Fig. 4:**
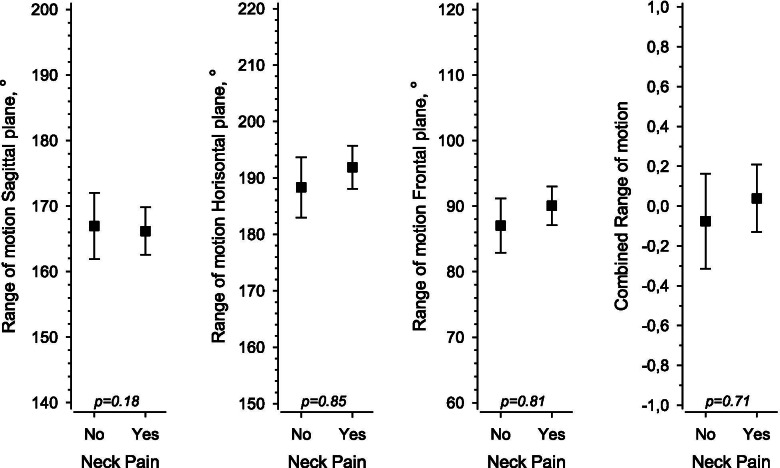
Passive range of motion in the subjects with no neck pain (No) and those with occasional or chronic neck pain (Yes) during the 16-year follow-up. The combined range of motion values have been calculated from the mean of the standardized of range of motion values in the sagittal, horizontal, and frontal planes. Boxes show means and whiskers indicate 95% confidence intervals

The mean (95% CI) range of motion for passive mobility of the cervical spine in the sagittal plane, frontal plane, and horizontal plane was 167 (162 to 172)^0^, 87 (83 to 91)^0^, and 188 (183 to 193)^0^, respectively, in the No pain group and 166 (162 to 170)^0^, 90 (87 to 93)^0^, and 192 (188 to 192)^0^, respectively, in the Pain group (Fig. 4). No statistically significant differences between groups were observed in the sagittal plane (*p* = 0.18), horizontal plane (*p* = 0.85), frontal plane (*p* = 0.81) or combined range of motion (*p* = 0.71) values (age-adjusted *p* values, Fig. 4). The range of motion values of the Chronic group did not differ from those of the No pain (*p* = 0.06 to 0.29) or Occasional group (*p* = 0.21 to 0.54).

## Discussion

This study explored whether neck muscle isometric strength or cervical spine mobility are associated with future neck disability in working-age women with no neck pain at baseline. As far as we know, this is the first study to investigate the association of neck muscle strength with spine mobility and neck disability using measurements of neck muscle strength and cervical spine mobility recorded as many as 16 years earlier. Thus, this study provides valuable information on the topic. The results indicate that the later occurrence of neck disability is not associated with either neck muscle strength or passive mobility of the cervical spine.

Although chronic neck pain and related disability is a common complaint, only a few studies have investigated the correlations of perceived neck-related disability with different physical capacity measures, including the use of linear regression analysis. Among the few correlation studies with neck-related disability as an outcome, Saavedra-Hernandez et al. [37] found a weak but significant negative correlation (r = − 0.18, *p* = 0.04) between cervical extension range of motion and disability in patients with chronic neck pain. Rudolfsson et al. [38], in turn, found that extension in the upper cervical levels and flexion in the lower levels were reduced in people with chronic neck pain. In addition, many studies with chronic neck pain as an outcome have reported that patients with chronic neck pain present reduced cervical range of motion [21, 24, 39, 40] and weaker maximal isometric strength of neck muscles compared to healthy controls [11–15, 39]. Lower neck muscle strength may be due to inhibition of maximal muscle contraction caused by pain. However, it should be noted that the above-mentioned studies observed the relationship at the same time point in time, whereas we investigated the relationship many years later. To our knowledge, very few studies have looked at the association between muscle strength and/or mobility and future pain/disability in the neck region after a lengthy period of time. Timpka et al. [41], in a large cohort study with a 17-year follow-up, found that low overall isometric muscle strength in youth was not associated with the development of musculoskeletal pain in adulthood in the neck/shoulders, back/hips or arms/legs. According to the systematic review by Hamberg-van Reenen et al. [42], similar results have been reported for other regions of the body, implying that no association exists between trunk muscle strength, muscle endurance or mobility of the lumbar spine and the risk of future low back pain.

In an earlier study on 192 women from the same study population, the occurrence of neck pain after 6 years was not predicted by either neck muscle strength or cervical spine passive mobility [27]. However, in that study we did not ask about the occurrence of chronic neck pain or use the NDI as an outcome measure. The participants were only asked whether they had experienced neck pain since the baseline measurements 6 years earlier and how many days in total they had experienced neck pain during the preceding 12 months. In the present study, the prevalence of chronic neck pain was 3%, which is somewhat lower than the approximately 5% previously reported globally [6, 7, 29]. Moreover, the neck disability index in most of these subjects was low. This is due to the study design, as the subjects were initially healthy adults with no history of neck pain.

Given our present findings and the previous literature it would seem plausible that factors other than cervical mobility and muscle strength predict neck disability. Sihawong et al. [43], in a one-year prospective cohort study in office workers, found that frequent neck extension during the workday and a high body mass index predicted chronic neck pain. Further, they found that high initial pain intensity and high psychological job demands were also predictors of chronic neck pain. In another one-year prospective study in office workers, Jun et al. [44] found that older age, increased sedentary hours, higher job strain, and stress were risk factors for the development of neck pain. Furthermore, physical factors, such as impaired in neck muscle endurance [39] and imbalance in sagittal alignment [45], female gender [46] and comorbidities [47, 48], have been reported to be important predictors of neck disability. Cross-sectional studies, in turn, indicate that psychosocial factors such as depression, anxiety, and catastrophization may be predisposing factors for the development of chronic neck pain, as defined by the Neck Disability Index [49–51]. One predisposing factor alone, however, does not necessarily lead to chronic neck pain. Instead, a combination of predisposing factors needs to be present to trigger neck pain, and such combinations most likely vary between individuals [47].

The strengths of this study are the length of the follow-up and low drop-out rate. The study also has its limitations. The study population in the chronic group was too small to permit analysis of whether neck strength and mobility are associated with the development of chronic neck pain. Moreover, the age range of the participants varied by 40 years, which could put them in different risk categories for several health-related reasons. However, this confounding factor was to some extent overcome by using an age-adjusted regression model. In addition, we had no information on whether the present participants exercised regularly following the baseline measurements, and if so, whether this was associated with any prevalence of neck pain 16 years later. Lastly, we do not know whether these results apply to aging populations. Some studies suggest that neck strength declines with age [52, 53] and that this decline is associated with neck pain [11–16]. Thus, to investigate this study topic thoroughly, further prospective longitudinal research with a larger cohort of healthy adults across the age spectrum and a variety of confounding factors in addition to neck muscle strength and mobility is required.

## Conclusion

This study found no evidence that isometric neck muscle strength or passive mobility of the cervical spine are associated with the later occurrence of neck pain or disability among originally pain-free women. Therefore, screening healthy subjects for weaker neck muscle strength or poorer cervical spine mobility alone cannot be recommended for preventive purposes. In addition to these physical capacity measures, future follow-up surveys should also include other long-term predictors such as psychosocial and work-related physical risk factors to predict neck pain and related disability.

## Data Availability

The datasets generated and analyzed during the current study are available from the corresponding author on reasonable request.

## Abbreviations

CMS: Cervical measurement system
CI: Confidence interval
IASP: The International Association for the Study of Pain
ICC: Intraclass correlation coefficient
NDI: Neck Disability Index
NSMS: Neck strength measurement system
PROM: Passive range of motion
SD: Standard deviation
VAS: Visual analogue scale
